# Gold Nanoparticles with *N*‐Heterocyclic Carbene/Triphenylamine Surface Ligands: Stable and Electrochromically Active Hybrid Materials for Optoelectronics

**DOI:** 10.1002/advs.202400752

**Published:** 2024-05-22

**Authors:** Ningwei Sun, Shivam Singh, Haoran Zhang, Ilka Hermes, Ziwei Zhou, Hendrik Schlicke, Yana Vaynzof, Franziska Lissel, Andreas Fery

**Affiliations:** ^1^ Leibniz‐Institut für Polymerforschung Dresden e.V. Hohe Straße 6 01069 Dresden Germany; ^2^ Chair for Emerging Electronic Technologies Technical University of Dresden Nöthnitzer Str. 61 01187 Dresden Germany; ^3^ Leibniz Institute for Solid State and Materials Research Dresden Helmholtzstraße 20 01069 Dresden Germany; ^4^ Hamburg University of Technology Kasernenstraße 12 21073 Hamburg Germany; ^5^ Chair for Physical Chemistry of Polymeric Materials Technische Universität Dresden Bergstraße 66 01069 Dresden Germany

**Keywords:** electrochromism, gold nanoparticles, hole‐transport materials, hybrid materials, N‐heterocyclic carbenes, triphenylamines

## Abstract

Organic‐hybrid particle‐based materials are increasingly important in (opto)electronics, sensing, and catalysis due to their printability and stretchability as well as their potential for unique synergistic functional effects. However, these functional properties are often limited due to poor electronic coupling between the organic shell and the nanoparticle. *N*‐heterocyclic carbenes (NHCs) belong to the most promising anchors to achieve electronic delocalization across the interface, as they form robust and highly conductive bonds with metals and offer a plethora of functionalization possibilities. Despite the outstanding potential of the conductive NHC‐metal bond, synthetic challenges have so far limited its application to the improvement of colloidal stabilities, disregarding the potential of the conductive anchor. Here, NHC anchors are used to modify redox‐active gold nanoparticles (AuNPs) with conjugated triphenylamines (TPA). The resulting AuNPs exhibit excellent thermal and redox stability benefiting from the robust NHC‐gold bond. As electrochromic materials, the hybrid materials show pronounced color changes from red to dark green, a highly stable cycling stability (1000 cycles), and a fast response speed (5.6 s/2.1 s). Furthermore, TPA‐NHC@AuNP exhibits an ionization potential of 5.3 eV and a distinct out‐of‐plane conductivity, making them a promising candidate for application as hole transport layers in optoelectronic devices.

## Graphical abstract

An organic‐hybrid material is prepared using robust and conductive *N*‐heterocyclic carbene to couple gold nanoparticles and triphenylamines. The redox‐active nanoparticles are stable and show fast red‐to‐green electrochromic response. Furthermore, their energy levels and conductivity make them a model for designing new hole‐transport layers for emerging photovoltaics.

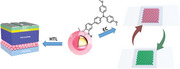

## 1 Introduction

Coupled inorganic–organic nanostructures offer breakthroughs for a range of applications ranging from fields as diverse as photovoltaics, spin memories, or optical upconverters to sensing or catalytic applications.^[^ ^1^ ^]^ Nanoparticles exhibit unique properties that are not accessible at the macroscale, such as the quantum size effect for semiconducting particles and localized surface plasmon resonance in the case of noble metal nanoparticles. Elevating them to hybrid particle‐based materials combines the merits of the inorganic (nanoparticle) with the merits of organic (molecules) and further adds beneficial synergetic effects. New devices and components^[^ ^2^ ^]^ will widely be based on assemblies instead of individual nanoparticles.^[^ ^3^ ^]^ However, the key challenge for this material class is facilitating an electronic delocalization across the metal–organic interface. Classical coupling of gold nanoparticles relies on thiol‐Au bonds. Because thiol‐Au bonds can be efficiently formed by exchanging weaker binding amine^[^ ^4^ ^]^ or citrate^[^ ^5^ ^]^ ligands on gold surfaces, they have been extensively utilized, among other for the attachment of various biomolecules to gold nanoparticles^[^ ^6^ ^]^ or to form hybrid materials of gold nanoparticles and conjugated, sulfur‐based polymers, which are promising materials for printable conductors.^[^ ^7^ ^]^ In this regard, also sintering‐free inks from gold nanorods and polythiophenes with high conductivities were reported.^[^ ^8^ ^]^ While the above examples underline that sulfur‐gold interactions offer a versatile method to create of gold nanoparticle/molecular interfaces, the linkage still possesses inherent drawbacks. On the one hand, due to their chemical nature, thiols are prone to oxidation,^[^ ^9^, ^10^ ^]^ potentially leading to changes or degradation of device performance over time. On the other hand, it was reported that the electronic structure of thiol‐gold bonds leads to inferior electronic interfaces,^[^ ^11^, ^12^ ^]^ which is especially problematic when aiming at the development of hybrid, particle‐based materials for applications requiring efficient charge transport. Dithiocarbamates have been discussed as an alternative^[^ ^11^ ^]^ and sp hybridized carbons were shown to facilitate a strong electronic coupling to gold electrodes.^[^ ^13^, ^14^ ^]^ However, to access the high application potential of versatile and highly configurable nanoparticle‐based materials, reliable and reproducible chemical approaches for improving the classical nanoparticle/molecular interfaces are still lacking.

This is addressed in the present work by utilizing *N*‐heterocyclic carbenes to bind functional molecules to gold nanoparticles. NHCs have emerged as one of the most promising functional groups to anchor ligands on metal surfaces or nanoparticles. Their chemical structure is easily modulated, they form highly stable NHC–metal bonds, and furthermore offer unique electronic properties.^[^ ^15^, ^16^, ^17^, ^18^, ^19^ ^]^ Interestingly, although NHCs have been used extensively as ligands for metal nanoparticles,^[^ ^20^, ^21^, ^22^, ^23^, ^24^, ^25^, ^26^ ^]^ so far the goal was mainly to improve colloidal stability. Only few examples explore the covalent NHC‐metal bonds to promote charge transfer across the metal–organic interface.^[^ ^27^, ^28^, ^29^ ^]^ However, utilizing this aspect would allow to create truly hybrid nanomaterials, where synergistic effects lead to enhanced functionality beyond those of the singular components. So far, the study of the emergent electronic and optoelectronic properties of such systems remains very much in its infancy, limiting the ability to rationally design new nanomaterials with properties tailored toward specific applications. In‐depth studies of NHC‐coupled metal nanoparticles and ligands with a second functionality will not only help understanding the interesting electronic properties of NHC structures, but also open a range of applications for metal nanoparticles, which normally are coated with insulated ligands, making them not suited for electrical devices.

For example, such systems carry high promise for the field of optoelectronics, in which a suitable highest occupied molecular orbital (HOMO) and ideally an intrinsically high hole mobility are necessary. Triphenylamine (TPA) based materials have been widely used as hole transport materials (HTMs) in organic light‐emitting diodes (OLEDs), organic solar cells (OSCs), and organic field‐effect transistors (OFETs).^[^ ^30^, ^31^, ^32^ ^]^ In TPA, an amine carries three phenyl groups via sp^2^ hybridized bonds. Due to the strong electron‐donating properties, good thermal and morphological stability, high hole‐transport capabilities can be achieved. For example, a representative TPA‐based HTM, spiro‐OMeTAD, is the most widely employed HTM in perovskite‐based solar cells (SCs).^[^ ^33^, ^34^, ^35^, ^36^, ^37^, ^38^ ^]^ However, in its pristine form, spiro‐OMeTAD has a low mobility due to the large intermolecular distance caused by the propeller‐like structures of TPA, making the use of dopants indispensable. Various molecular designs of TPA‐based HTMs have been investigated to increase the intermolecular charge transport of TPA and to realize an intrinsically high hole mobility without sacrificing the stability of the SCs.^[^ ^39^, ^40^, ^41^, ^42^, ^43^ ^]^ In addition, TPAs can be easily oxidized and show strong and vibrant color changes during the redox process.^[^ ^44^ ^]^ The introduction of electron‐donating methoxy groups (OME) into the para‐position of TPA units can effectively prevent the irreversible coupling to form TPA dimer, and further reduce the oxidation potential of the TPA, making the redox process even more stable.^[^ ^45^, ^46^ ^]^ Various TPA‐based electrochromic materials have been designed and applied in high‐performance electrochromic devices.^[^ ^47^, ^48^, ^49^, ^50^, ^51^ ^]^ In addition to the introduction of organic functional groups, the hybridization of organic electrochromic materials and inorganic nanomaterials – e.g., AuNPs – can allow to synergistically combine their merits and improve the electrochromic performance: e.g., a fast‐switching speed can be realized due to the reduced charge transfer resistance.^[^ ^52^, ^53^, ^54^ ^]^ However, there is generally a lack of sufficiently strong interactions between the organic and inorganic components. As a consequence, the organic and inorganic components are not coupled well enough to result in synergistic effects, and the electrochemical properties of organic materials are not influenced by the inorganic nanoparticles.

**Figure 1:**
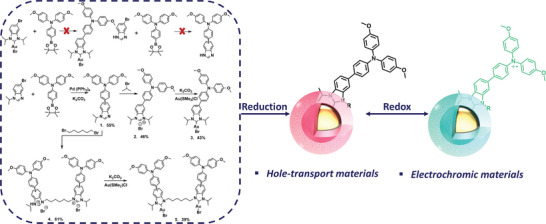
Synthetic route to redox‐active hybrid gold nanoparticles TPA‐NHC@AuNP and TPA‐BiNHC@AuNP.

Herein, by careful molecular design, we present two kinds of TPA‐NHC@AuNP, where a metal core and an organic redox‐active moiety are coupled via an NHC anchor (**Figure** **1**). The robust and conductive NHC‐gold bond is boosting the thermal and electrochemical stability as well as the charge transport of the resulting nanocomposites. Utilizing an OME group further adds to the redox‐stability of the material, enhancing the potential for applications in electrochromic devices. In addition, the NHC‐coupled AuNP/TPA system could be a model for designing new hole‐transport layers for emerging photovoltaics, as the AuNPs improve charge transport.

## 2 Results and Discussion

In comparison with other cross coupling reactions, Suzuki couplings need fewer toxic reagents and can furthermore be carried out under mild reaction conditions. However, adopting the reaction to the coupling of the NHC moieties with the TPA‐OME units required to consider several restraints. When seeking to directly couple bromo‐NHC‐Au (I) with 4‐methoxy‐*N*‐(4‐methoxyphenyl)‐*N*‐(4‐(4,4,5,5‐tetramethyl‐1,3,2‐dioxaborolan‐2‐yl)phenyl)aniline (TPA‐OME‐based boronic ester), the coupling reaction failed, probably due to the interaction between the NHC‐Au (I) and the platinum catalyst used in the Suzuki reaction. Starting with 5‐bromo‐1*H*‐benzoimidazole to couple TPA‐OME‐based boronic ester was also problematic, as the protic proton on the imidazole can also react with TPA‐OME‐based boronic ester, making the separation very difficult. To circumvent this problem, we first protected the protic proton of 5/6‐Bromo‐1‐isopropyl‐benzimdazole by introducing propane. This allowed us to then couple the 5/6‐bromo‐1‐isopropyl‐benzimdazole to the TPA‐OME‐based boronic ester via Suzuki reaction. (see Supporting Information for detailed information), yielding compound **1** (Figure 1). The following alkylation with 2‐bromopropane afforded the corresponding benzimidazolium salt **2**. To further increase the stability, we also synthesized a bidentate NHC ligand using a hexane to link two NHCs. Here, alkylation of **1** with 1,6‐dibromohexane affords the corresponding benzimidazolium salt **4**. Then the two target NHC‐Au(I) complexes **3** and **5** were synthesized by reacting the benzimidazolium salts **2** and **4** with Au(SMe_2_)Cl in the presence of K_2_CO_3_. ^1^H and ^13^C NMR characterizations documented the successful synthesis of these compounds (Figures S1–S7, Supporting Information).

**Figure 2:**
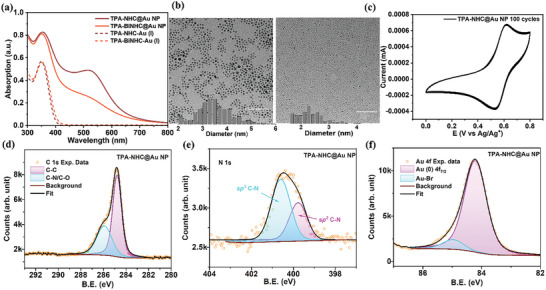
a) UV–vis absorption of the NHC‐Au(I) complexes and the resulting TPA‐NHC@Au NP and TPA‐BiNHC@Au NP. b) TEM images of TPA‐NHC@Au NP (left) and TPA‐BiNHC@Au NP (right) (scale bar: 50 nm). c) CV scans of TPA‐NHC@Au NP for 100 cycles. d) C1s, e) N 1s, and f) Au 4f XPS core level spectra of TPA‐NHC@Au NP.

AuNPs functionalized with TPA‐OME via NHC anchors were then synthesized by a direct reduction of the corresponding NHC‐Au(I) complexes using NaBH_4_ as the reducing agent. The size of monodentate AuNP (named as TPA‐NHC@AuNP) was found to be ≈3.5 nm, while the size of the bidentate AuNP (named as TPA‐BiNHC@AuNP) was found to be ≈2.2 nm. The smaller size of TPA‐BiNHC@AuNP might be attributed to the larger ligand structure, which potentially hinders the growth of the nanoparticles. The optical properties of the AuNPs were investigated by UV–vis spectroscopy (**Figure** **2**a). The absorption at ≈535 nm is associated from the plasmonic resonance of the AuNPs, while the absorption at ≈360 nm is attributed to the π–π* transitions of the aromatic NHC‐TPA‐OME moieties. As expected, the plasmonic absorption of the bidentate NP is weaker than that of the monodentate NP due to the smaller size. Despite the smaller size, the TPA‐BiNHC@AuNP showed a better stability: When heating the NPs in boiling chloroform for 2 h, the monodentate TPA‐NHC@AuNP aggregated with the plasmonic absorption reduced and broadened while the TPA‐BiNHC@AuNP only showed a small degree ripening with the plasmonic absorption narrowing and increasing slightly (Figure S10, Supporting Information). This is consistent with the higher stability of multidentate NHC ligands.^[^ ^25^, ^26^, ^27^ ^]^ It has to be noted that the stability, even for the monodentate variant, exceeds those of other common anchors, e.g., thiols, by far. A further improvement in stability might be realized by top‐down ligand exchange methods.^[^ ^27^ ^]^

X‐ray photoemission spectroscopy (XPS) was utilized to determine the chemical states/ composition of both TPA‐NHC@AuNPs synthesized in this report (Figure 2; Figures S8 and S9, Supporting Information). The C 1s core level spectrum of NHC‐AuNP can be deconvoluted into two components centered at 284.8 and 286.0 eV, corresponding to C─C and C─O/C─N bonds, respectively, as is shown in Figure 2d.^[^ ^55^ ^]^ The C 1s peak at 284.8 eV is used to calibrate the other core level spectra in order to eliminate charging effects. The N 1s spectrum is shown in Figure 2e and is also deconvoluted into two components at 399.8 eV and 400.6 eV, attributed to sp^3^ and sp^2^ C‐N bonding, respectively.^[^ ^56^ ^]^ The C1s and N 1s signals evidence the presence of the NHC ligands on the AuNPs. In addition, the Au 4f core level spectrum is also analyzed to investigate the bonding and oxidation stability of the TPA‐NHC@AuNPs and is shown in Figure 2f. The Au 4f spectrum shows a doublet located at 84.2 eV (Au 4f_7/2_) and 87.9 eV (Au 4f_5/2_) with a separation of 3.7 eV (Figure S8, Supporting Information) which is a characteristic of Au(0) NP. An additional species is deconvoluted at 85.0 eV (Figure 2f) and is attributed to the Au‐Br bond present in the TPA‐NHC@AuNPs.^[^ ^57^ ^]^ There is no evidence of oxidation of Au, which would be expected to present either at a difference of ≈2.0 eV for Au(I) or 3.3 eV for Au(III) compared to the Au(0) peak.^[^ ^55^, ^58^ ^]^

The electrochemical behavior of the TPA‐NHC@AuNPs was studied by cyclic voltammetry (CV) measurements in an electrochemical cell with 10^−4^ M of NPs, and 0.1 m of TBABF_4_ in anhydrous DCM as supporting electrolyte, Ag/Ag^+^ as the reference electrode and a platinum plate as the working electrode. As shown in Figure 2c, the AuNP exhibited a high electrochemical stability in continuous 100 scans. The excellent stability can be attributed to the electron‐donating OME groups could effectively lower the oxidation potential and obtain stable radical cations without oxidative coupling reactions. Furthermore, after the CV scans, the absence of obvious color/absorption changes indicate the excellent stability of the AuNPs without aggregation or decomposition, which can be attributed to the robust NHC‐gold bond. Based on the promising results regarding the electrochemical stability, the electrochromic properties of the AuNP were investigated. Before fabricating the electrochromic devices, the absorption/color changes of TPA‐NHC@AuNP were studied using chemical oxidation: Upon the addition of copper perchlorate, the absorption at 330 nm gradually decreased, while the absorption at ≈760 nm increased (Figure S11, Supporting Information). The color of the NP solution changed from red to dark green. The plasmonic absorption of NP did not decrease, indicating the good stability of the NP during oxidation.

**Figure 3:**
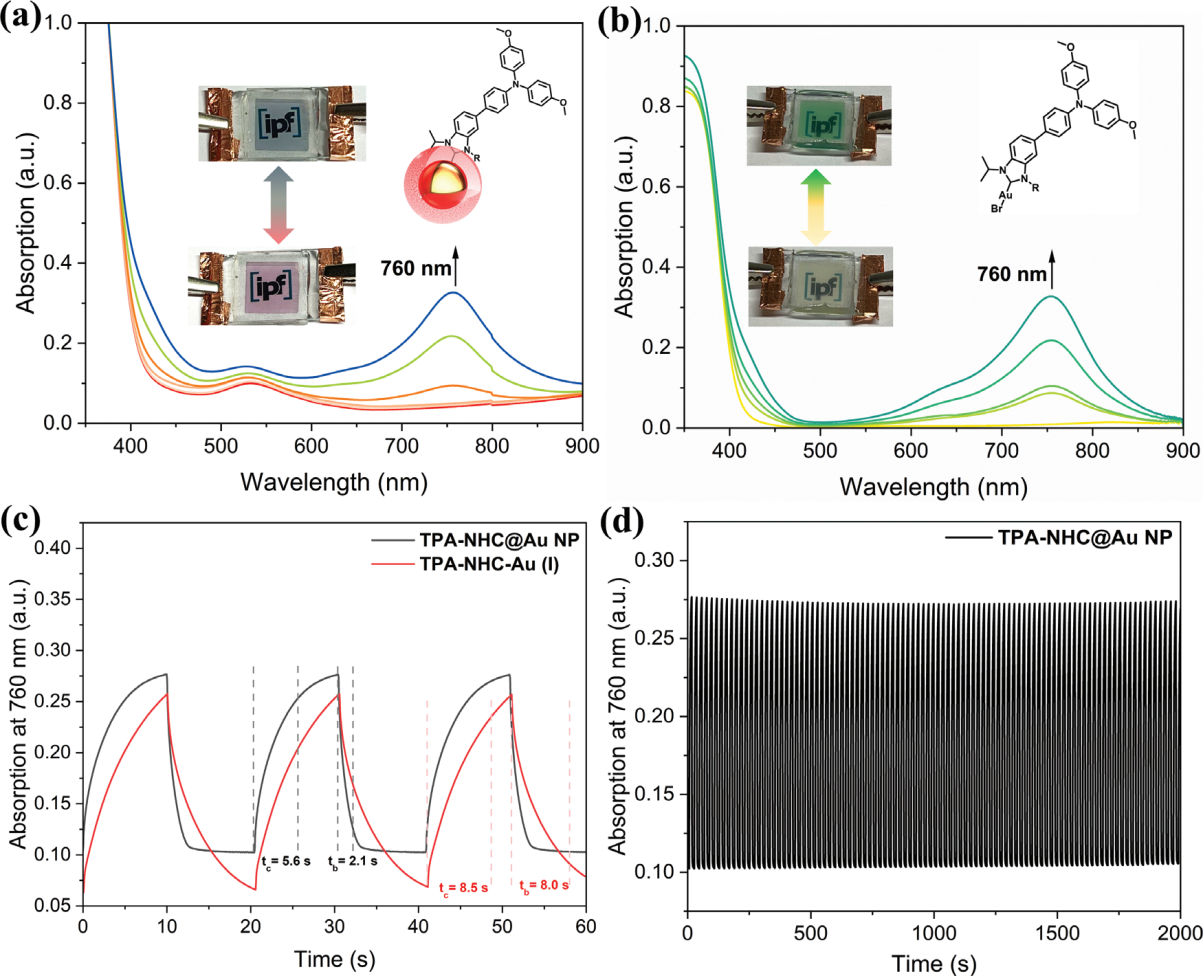
a) Absorption changes of the TPA‐NHC@AuNP‐based electrochromic device upon oxidation (inset: the color changes of the device). b) Absorption changes of the NHC‐Au(I)‐based electrochromic device upon oxidation (inset: the color changes of the device). c) Switching speed comparison of the electrochromic devices using TPA‐NHC@AuNP and NHC‐Au(I) as the active materials. d) Switching stability of the TPA‐NHC@AuNP‐based electrochromic device for 100 cycles.

Then a gel‐type electrochromic device composed of ≈0.1 µmol NPs and 1 µmol tetrabutylammonium tetrafluoroborate (TBABF_4_) as the supporting electrolyte in propylene carbonate (PC) with 2 mg poly(methyl methacrylate) (PMMA) was fabricated. For comparison, the electrochromic device using NHC‐Au(I) **3** as the active material was also fabricated. The spectroelectrochemical behaviors of both materials were investigated as shown in **Figure** **3**a,b. The two devices showed similar absorption changes, except that the device containing NPs additionally showed an inherent plasmonic absorption. For both materials, when applying positive potentials, a new absorption at ≈760 nm increased. The color of the device composed of TPA‐NHC@AuNP changed from red to dark green, while the color of the device composed of the NHC‐Au(I) complex changed from colorless to green.

Electrochromic switching studies were carried out to investigate the devices more deeply. Here, the absorption at 760 nm was monitored as a function of time by stepping the potentials between the oxidized states and neutral states. Figure 3c demonstrates the electrochromic switching behavior of the devices, with the response time calculated at 90% of the full optical changes. The response time (coloring/bleaching) of TPA‐NHC@AuNP and NHC‐Au(I) complex were calculated to be 5.6 s/2.1 s, and 8.5 s/8.0 s, respectively. The higher response speed of TPA‐NHC@AuNP can be attributed to the improved charge transfer achieved by including AuNPs via an electronically delocalized NHC anchor. Faster response speed can be realized by the optimization of the gel‐type electrochromic devices. Moreover, the electrochromic device exhibited excellent switching stability, keeping the color/absorption changes without decay for over 1000 cycles (Figure S12, Supporting Information).

**Figure 4:**
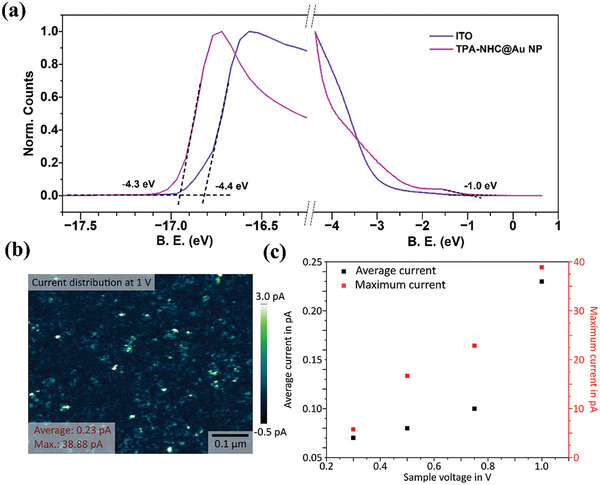
a) Secondary electron cut‐off and valence band region of TPA‐NHC@AuNPs characterized by UPS. b) C‐AFM images under the bias of 1 V. c) Maximum and average C‐AFM currents measured at the bias of 300, 500, 750, and 1000 mV.

NHC‐Coupled AuNP/TPA systems could be an interesting model for designing new hole‐transport layers for emerging photovoltaics, as the coupling with AuNPs improves the charge transport of the ubiquitously used TPA. To investigate the potential of such NHC‐coupled AuNP/TP hybrid materials as hole transport layers in optoelectronic devices, we characterized the energetics and out‐of‐plane conductivity of the TPA‐NHC@AuNP system. The HOMO level of TPA‐NHC@AuNPs spin‐coated on ITO substrate was measured by ultra‐violet photoemission spectroscopy (UPS). **Figure** **4**a represents the secondary electron cut‐off and valence band region of the TPA‐NHC@AuNP. The hybrid particles exhibit a work‐function of 4.3 eV and a valence band onset of 1.0 eV, corresponding to a HOMO level of −5.3 eV. This HOMO value matches well with that of other HTMs such as poly[bis(4‐phenyl)(2,4,6‐trimethylphenyl)amine] (PTAA) or self‐assembled molecules (SAMs) that are commonly used in optoelectronic devices.^[^ ^59^, ^60^, ^61^, ^62^, ^63^ ^]^

A density function theory (DFT) calculation using the model of TPA‐NHC‐Au (0) was carried out to study the electronic coupling between the AuNP and TPA unit (Figure S15, Supporting Information). In the HOMO and LUMO orbits, the electron can be delocalized over Au atom and conjugated NHC‐TPA moiety, indicating a highly conductive NHC‐Au bond. To evaluate the electronic coupling in TPA‐NHC@AuNP, we performed conductive atomic force microscopy (C‐AFM) measurements and captured the out‐of‐plane current distribution of a spin‐coated film on ITO at different sample voltages. At a sample voltage of 1 V (Figure 4b, respective topography data in Figure S13, Supporting Information), we measured an average and maximum current of 0.23 and 38.88 pA, respectively. Reducing the voltage to 750, 500, and 300 mV resulted in a monotonic decrease of the detected average and maximum current (Figure 4c, C‐AFM images in Figure S14, Supporting Information). Taken together, the energetics and conductivity measurements suggest that the hybrid material shows potential for an application as HTM. As a preliminary test, we fabricated proof‐of‐concept inverted triple cation perovskite‐based SCs utilizing TPA‐NHC@AuNPs as HTM. The device structure used to fabricate SCs is shown in Figure S16a (Supporting Information).^[^ ^64^ ^]^ Detailed information regarding the solution preparation and device fabrication/characterization is given in the Supporting Information. Figure S16b (Supporting Information) shows the illuminated current density–voltage (J–V) characteristic of a champion device with TPA‐NHC@AuNPs as HTL. A power conversion efficiency (PCE) of 8.79% was achieved, which is lower than that of standard HTMs, but still shows the promise of TPA‐NHC@AuNPs for future applications. The average PCE for TPA‐NHC@AuNP HTL‐based PSCs over 16 devices is shown in Figure S17 (Supporting Information). This is a preliminary result, and we believe that higher efficiencies can be achieved by optimization of the processing conditions of TPA‐NHC@AuNPs or by further modifying the chemical structure of the TPA ligand and the size of the AuNPs.

## 3 Conclusion

In summary, with careful molecular designs using the Suzuki coupling, we present two new hybrid gold nanoparticles (AuNPs) systems carrying electronically functional triphenylamine (TPA) ligands bound via stable and conductive *N*‐heterocyclic carbene (NHC) anchors. Extensive characterization by NMR, TEM and XPS were carried out to probe the properties of the resulting AuNPs. Both systems show a thermal stability exceeding those of thiol protected AuNPs. The bidentate TPA‐BiNHC@AuNP are characterized by a smaller size, but a higher thermal stability compared to the monodentate variant TPA‐NHC@AuNP. Benefiting from the robust NHC‐Au bond as well as the para‐protection of the OME group, the TPA‐NHC@AuNP exhibited a high electrochemical stability. TPA‐NHC@AuNP‐based electrochromic devices exhibited strong color changes from red to dark green with a high switching stability over 1000 cycles. In comparison to the NHC‐Au(I) complex, TPA‐NHC@AuNP exhibited a much faster response speed due to the nanoparticles and the improved charge transfer between AuNP and the TPA unit. Moreover, the HOMO energy level measured by UPS is well‐matched with that of other HTMs used in optoelectronic devices such as solar cells. While our preliminary tests examining the use of TPA‐NHC@AuNP as HTM in perovskite solar cells showed it is – at present – inferior to well investigated HTMs, the results suggest that it is a promising system for further studies. Optimization efforts to improve the performance of the devices are currently underway in our lab. In comparison to conventional physisorbed or thiol based coupled core‐shell particles, the electronic delocalization across the metal‐organic interface facilitates a strong electronic coupling, while at the same time maintaining the advantages of a system that can be processed from solution and is colloidally stable.

## Conflict of Interest

The authors declare no conflict of interest.

## Supporting information

Supporting Information

## Data Availability

The data that support the findings of this study are available from the corresponding author upon reasonable request.
