# Supplementary material for: Gold Nanoparticles with N‐Heterocyclic Carbene/Triphenylamine Surface Ligands: Stable and Electrochromically Active Hybrid Materials for Optoelectronics
Source: Adv Sci (Weinh). 2024 May 22;11(29):2400752. doi: 10.1002/advs.202400752 (PMC11304275; doi:10.1002/advs.202400752)
Supplement: Supplementary file 1 — Supporting Information [file ADVS-11-2400752-s001.pdf]

ADVANCED SCIENCE
Open Access

Supporting Information

for *Adv. Sci.*, DOI 10.1002/advs.202400752

Gold Nanoparticles with *N*-Heterocyclic Carbene/Triphenylamine Surface Ligands: Stable and Electrochromically Active Hybrid Materials for Optoelectronics

Ningwei Sun, Shivam Singh, Haoran Zhang, Ilka Hermes, Ziwei Zhou, Hendrik Schlicke, Yana Vaynzof, Franziska Lissel and Andreas Fery**

Gold Nanoparticles with N-Heterocyclic Carbene/Triphenylamine Surface Ligands: Stable and Electrochromically Active Hybrid Materials for Optoelectronics

Ningwei Sun[1], Shivam Singh[2], Haoran Zhang[1], Ilka Hermes[1], Ziwei Zhou[1], Hendrik Schlicke[1], Yana Vaynzof [2,4], Franziska Lissel,[1,3], Andreas Fery[1,5*]*

[1]Leibniz-Institut für Polymerforschung Dresden e.V., Hohe Straße 6, Dresden 01069, Germany

[2]Chair for Emerging Electronic Technologies, Technical University of Dresden, Dresden, Germany

[3]Hamburg University of Technology, Kasernenstraße 12, 21073 Hamburg, Germany

[4]Leibniz Institute for Solid State and Materials Research Dresden, Dresden, Germany

[5]Chair for Physical Chemistry of Polymeric Materials, Technische Universität Dresden, Bergstraße66, 01069 Dresden, Germany

Table of Contents

1. Experimental Methods and Instrumentation 2
   Nuclear Magnetic Resonance (NMR) Spectroscopy 2
   Ultraviolet-visible (UV/Vis) Spectroscopy 2
   Cyclic voltammetry (CV) 2
   Transmission Electron Microscopy (TEM) 3
   Scannning Electron Microscopy (SEM) 3
   Conductive Atomic Force Microscopes (C-AFM) 3
   Fabrication of electrochromic devices 3
   Spectroelectrochemistry 4
   X-ray and ultra-violet Photoemission spectroscopy (XPS and UPS) 4
   Materials and Solution Preparation for Solar Cell Experiments 4
   Device Fabrication and Characterization for Solar Cell Experiments: 5
2. Synthesis protocols of the TPA-NHC-Au (I) complexes 5
   Synthesis of 5/6-(4-(bis(4-methoxyphenyl)amino)phenyl)-1-isopropyl-benzimidazole (1): 5

Synthesis of 5-(4-(bis(4-methoxyphenyl)amino)phenyl)-1,3-diisopropyl-benzimidazolium bromide (2): 6

Synthesis of (5-(4-(bis(4-methoxyphenyl)amino)phenyl)-1,3-diisopropyl-benzimidazolium)gold bromide (3):7

Synthesis of 1,6-di(5/6-(4-(bis(4-methoxyphenyl)amino)phenyl)-1,3-diisopropyl-benzimidazolium bromide)-hexane (4): 7

Synthesis of 1,6-di(5/6-(4-(bis(4-methoxyphenyl)amino)phenyl)-1,3-diisopropyl-benzimidazole)gold bromide)-hexane (5): 8

Synthesis of gold nanoparticles with *N*-Heterocyclic Carbene Modified with Triphenylamines as Ligands (NHC-TPA@Au NP): 9

3. Experimental Data 9

Nuclear Magnetic Resonance (NMR) Spectroscopy 9

XPS: 13

Thermal stabilities: 14

Chemical oxidation: 15

Long-term electrochromic switching stability: 15

AFM and C-AFM: 16

DFT calculation: 17

UPS and solar cells: 18

1. Experimental Methods and Instrumentation

Nuclear Magnetic Resonance (NMR) Spectroscopy

A Bruker Avance III 500 spectrometer was used to record ^{1}H NMR spectra at 500 MHz and ^{13}C NMR spectra at 126 MHz. The spectra were referenced to the residual solvent signals ($CDCl_3$: $\delta(^{1}H) = 7.26$ ppm, DMSO-d_6: $\delta(^{1}H) = 2.50$ ppm). The following abbreviations were used for ^{1}H NMR spectra data as listed: s - singlet, d - doublet, dd - doublet of doublet, t – triplet and m -multiplet.

Ultraviolet-visible (UV/Vis) Spectroscopy

UV-vis spectra were measured on UV/vis/NIR spectrophotometer Cary 5000 (Agilent Technologies Deutschland GmbH).

Cyclic voltammetry (CV)

Electrochemical analyses were carried out on the Autolab PGSTAT302N workstation. Cyclic

voltammetry (CV) measurements were carried out in a 3-electrode setup with platinum counter electrode, Ag/Ag+ reference electrode and platinum working electrodes. 0.1 M NaCl/HCl was used as the electrolyte. PANI@ NPs coated on ITO substrates were used as working electrodes.

Transmission Electron Microscopy (TEM)

TEM images were obtained using a Zeiss Libra 120 with an accelerating voltage of 120 kV. Samples were prepared by placing a 2 μL droplet of the diluted NP dispersions or the assembly film on TEM grids (Cu, 200 Mesh, coated with carbon film; Science Services GmbH).

Scannning Electron Microscopy (SEM)

The surface morphology of NP films was measured using a Zeiss SEM NEON 40 EsB CrossBeam in the in-lens mode with an accelerating voltage of 3.0 kV.

Conductive Atomic Force Microscopes (C-AFM)

C-AFM measurements on spin-coated NP films were performed in PeakForce TUNA mode on a Dimension Icon AFM (Bruker, USA). We used a PeakForce TUNA cantilever with a spring constant of k = 0.4 N/m and a conductive Ti/Au coating. In this measurement mode, the tip is oscillating with a low frequency of 1 kHz and the current is detected during the time of contact between tip and sample using a current amplifier. All measurements were performed consecutively with the same cAFM tip starting from low tip bias (0.3 V) to higher tip bias (1 V). Due to capacitive instabilities in the current amplifier, we performed a baseline correction of the current channel for all measurements. Moreover, the system features a 7.5 mV voltage offset which was subtracted from all measurement biases.

Fabrication of electrochromic devices

ITO glass (thickness of ITO ≈ 110 nm; resistance, 20 Ω / square) was cleaned by washing with Milli-Q water, acetone and isopropanol, respectively. The gel electrolyte was prepared by mixing poly(methyl methacrylate) (PMMA) powder (0.2 g) and tetrabutylammonium tetrafluoroborate (TBATFB) (32.9 mg) in 1 mL propylene carbonate, and then heated at 50 °C until the solution became clear. Then Au NPs (3 mg) in 0.2 mL $CHCl_3$ was added to the above gel electrolyte. The solvent was removed by vacuum evaporation to form a homogeneous gel. After that, the gel electrolyte containing Au NP was spread on ITO with a double-sided tape frame as the spacer, and another ITO substrate was covered on top, followed by sealing via a UV-curing adhesive. The effective electrochromic area

was about 1.2 cm^2.

Spectroelectrochemistry

Spectroelectrochemical properties were assessed by the combination of the electrochemical workstation (applying potentials) and the UV/vis/NIR spectrophotometer Cary 5000 (collecting the spectra with the range from 300 to 900 nm).

X-ray and Ultra-Violet Photoemission spectroscopy (XPS and UPS)

The samples were transferred to an ultrahigh vacuum chamber (ESCALAB 250Xi by Thermo Scientific, base pressure: 2×10^{-10} mbar) for XPS measurements. XPS measurements were carried out using an XR6 monochromated Al Kα source (hν = 1486.6 eV). A pass energy of 50 eV and 20 eV was used for survey and core level spectra, respectively. Ultraviolet photoelectron spectroscopy (UPS) measurements were carried out using a double-differentially pumped He discharge lamp ($h\nu = 21.22$ eV) with a pass energy of 2 eV and a bias at −5 V.

Materials and Solution Preparation for Solar Cell Experiments

All used reagents and solvents were purchased from commercial suppliers and used without further purification, unless noted otherwise. Perovskite films and devices were fabricated using PbI_2 and $PbBr_2$ (99.99% purity) purchased from TCI, organic halide salts were purchased from GreatCell Solar Materials, and cesium iodide (99.99% purity) was purchased from Alfa Aesar. The $PC_{60}BM$ was purchased from Luminescence Technology Corporation (Lumtec). The bathocuproine (BCP; sublimed grade, 99.99% purity) was purchased from Sigma-Aldrich. All the anhydrous solvents were purchased from Acros Organics.

The TPA-NHC@Au NP (used as hole transporting layer; HTL) were dissolved in anhydrous chloroform at room temperature inside the nitrogen filled glovebox with different concentrations: 10 mg/mL, 7 mg/mL and 3 mg/mL. The perovskite precursor solution (1.2 M) contained mixed cations (Pb, Cs, FA, and MA) and halides (I and Br) dissolved in a solvent mixture (DMF/DMSO = 4/1) according to a formula of $Cs_{0.05}(FA_{5/6}MA_{1/6})_{0.95}Pb(I_{0.85}Br_{0.15})_3$ with an excess of PbI_2 of 1%. The $PC_{60}BM$ (used as electron transport layer; ETL) was dissolved in anhydrous chlorobenzene with a concentration of 20 mg/mL and kept for overnight stirring at 70^0C inside the nitrogen filled glovebox. The BCP solution was dissolved in anhydrous isopropanol with a concentration of 0.5 mg/mL and kept for overnight stirring at 70^0C inside the nitrogen filled glovebox.

Device Fabrication and Characterization for Solar Cell Experiments:

Prepatterned ITO/glass substrates were sequentially cleaned with deionized water, acetone and 2-propanol (IPA) by ultrasonication for 10 min in each solvent. The ITO/glass substrates were then dried with N_2 and treated with oxygen plasma at 100 mW for 10 min. The TPA-NHC@Au NP solution was spin-coated over cleaned ITO/glass substrate at 4000 rpm for 30 seconds and annealed at 70^0C for 5 minutes on a hotplate inside a nitrogen filled glove box. The as-prepared HTL coated ITO substrates were transferred to drybox (relative humidity (RH) <1%) for perovskite deposition. The perovskite layer was deposited via a two-step spin-coating procedure with 1000 rpm for 10 s and 6000 rpm for 30 s. 150 µl of chlorobenzene was dripped on the spinning substrate during the last 5 seconds of the second spin-coating step. Subsequently, the samples were annealed at 100°C for 30 min. For the ETL deposition, the perovskite films are again transferred to the nitrogen filled glovebox. The $PC_{60}BM$ solution was spin-coated over the perovskite layer at 2000 rpm for 30 seconds (with a ramping speed of 1000 rpm/s) and kept for bench dry for 10 minutes. After that the BCP solution was spin-coated at 4000 rpm for 30 seconds (with a ramping speed of 1000 rpm/s) as hole-blocking layer. Finally, 80nm Ag was deposited under a vacuum of 4 X 10^{-7} mbar. The device area was defined as 4.5 mm^2 by metal shadow mask.

Current density-voltage (J-V) measurements were performed in ambient conditions under simulated AM 1.5 light with an intensity of 100 mW cm^{-2} (Abet Sun 3000 Class AAA Solar Simulator). The intensity was calibrated using a Si reference cell (NIST traceable, VLSI). Devices were scanned using a Keithley 2450 source measure unit from -0.5 to 1.2 V and back, with a step size of 0.05 V and a dwell time of 0.1 s. The pixel area was 3 mm by 1.5 mm.

2. Synthesis protocols of the TPA-NHC-Au (I) complexes

Synthesis of 5/6-(4-(bis(4-methoxyphenyl)amino)phenyl)-1-isopropyl-benzimidazole (1):

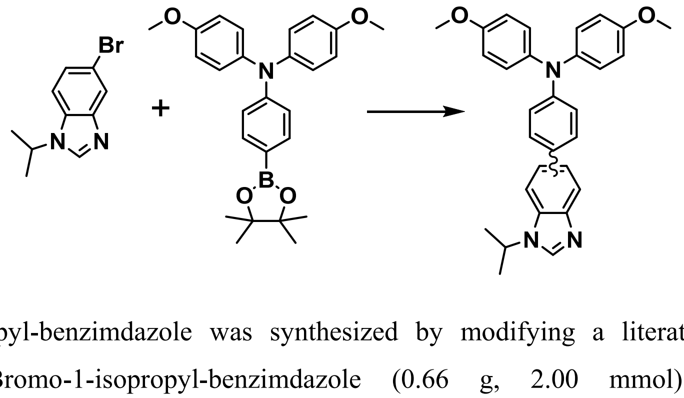

5/6-Bromo-1-isopropyl-benzimdazole was synthesized by modifying a literature protocol []. A mixture of 5/6-Bromo-1-isopropyl-benzimdazole (0.66 g, 2.00 mmol), 4-methoxy-N-(4-methoxyphenyl)-N-(4-(4,4,5,5-tetramethyl-1,3,2-dioxaborolan-2-yl)phenyl)aniline (0.88 g, 2.05 mmol), $Pd(PPh_3)_4$ (0.11 g, 0.1 mmol) and potassium carbonate (K_2CO_3, 2.8 g, 20.0 mmol) in 80 mL of

a degassed toluene/ethanol/water mixture (8:1:1 v/v/v) was stirred at 100 °C for 24 h under nitrogen atmosphere. After cooling to room temperature, the mixture was extracted with dichloromethane and washed successively with water (3 x 20 mL) and a brine solution (20 mL). After drying with anhydrous magnesium sulfate, the organic layer was filtered and evaporated under reduced pressure. The residue was purified by column chromatography (ethyl acetate/hexance=3:1) to obtain 0.91 g of yellow solid (55% yield). The product is a (roughly) 1:1 mixture of two isomers (5- and 6-substitution) that are difficult to separate by column chromatography. ^{1}H NMR (500 MHz, Chloroform-*d*) δ 8.15 (d, J = 16.4 Hz, 2H), 8.00 (s, 1H), 7.84 (d, J = 8.4 Hz, 1H), 7.58 – 7.49 (m, 4H), 7.46 (dt, J = 8.5, 3.6 Hz, 4H), 7.10 (d, J = 8.9 Hz, 8H), 7.04 – 6.97 (m, 4H), 6.90 – 6.80 (m, 8H), 4.77 – 4.58 (m, 2H), 3.81 (s, 12H), 1.66 (d, J = 6.7 Hz, 12H).

Synthesis of 5-(4-(bis(4-methoxyphenyl)amino)phenyl)-1,3-diisopropyl-benzimidazolium bromide (2):

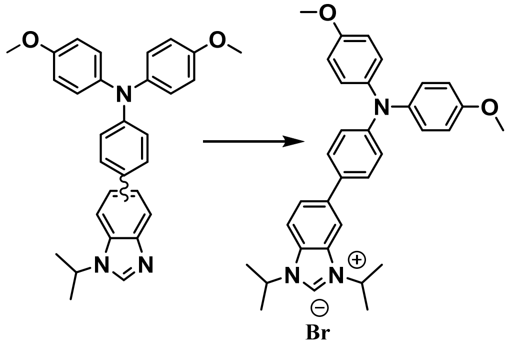

5/6-(4-(Bis(4-methoxyphenyl)amino)phenyl)-1-isopropyl-benzimidazole (0.40 g, 0.68 mmol, 1 eq.), 2-bromopropane (0.50 g, 4.1 mmol, 6 eq.), and 1 mL of tetrahydrofuran (THF) were mixed in a pressure vessel. The reaction was heated at 100 °C and allowed to proceed for 24 hours. After completion of the reaction, the mixture was carefully transferred to a 100 mL round bottom flask and the solvent, along with any unreacted 2-bromopropane, was removed by evaporation. The crude product was subjected to purification by column chromatography using a dichloromethane (DCM) and methanol (MeOH) mixture in a ratio of 20:1 as the eluent. The resulting white solid was collected with a yield of 46% (0.22 g). ^{1}H NMR (500 MHz, DMSO-d6) δ 9.75 (s, 1H), 8.27 (s, 1H), 8.15 (d, J = 8.8 Hz, 1H), 7.92 (d, J = 7.8 Hz, 1H), 7.66 (d, J = 8.7 Hz, 2H), 7.08 (d, J = 8.9 Hz, 4H), 6.96 (d, J = 8.9 Hz, 4H), 6.89 (d, J = 8.7 Hz, 2H), 5.11 (dp, J = 41.8, 6.7 Hz, 2H), 3.77 (s, 6H), 1.66 (d, J = 6.7 Hz, 12H).

Synthesis of (5-(4-(bis(4-methoxyphenyl)amino)phenyl)-1,3-diisopropyl-benzimidazolium)gold bromide (3):

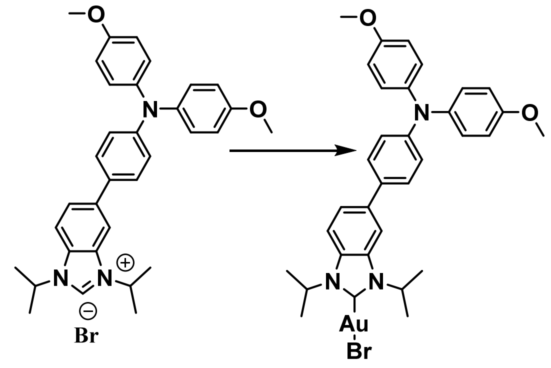

5-(4-(Bis(4-methoxyphenyl)amino)phenyl)-1,3-diisopropyl-benzimidazolium bromide (0.20 g, 0.26 mmol, 1 eq.), $Au(SMe_2)Cl$ (0.08 g, 0.28 mmol, 1.1 eq.), K_2CO_3 (0.18 g, 1.28 mmol, 5 eq.), and 5 mL of anhydrous acetone were added to a two-neck round flask. The mixture was then heated to 65 °C and stirred overnight to promote the formation of the desired product. After cooling to room temperature, the solvent was evaporated, and the residual solid was dissolved in chloroform and filtered over Celite. The solvent was removed under vacuum and the crude product was purified by column chromatography using a gradient eluent (from pure DCM to DCM : MeOH= 100 : 1) to yield a white solid. (0.09 g, yield: 43%) ^{1}H NMR (500 MHz, Chloroform-d) δ 7.70 (s, 1H), 7.64 (d, J = 8.6 Hz, 1H), 7.53 (d, J = 8.5 Hz, 1H), 7.38 (d, J = 8.5 Hz, 2H), 7.11 (d, J = 8.7 Hz, 4H), 7.01 (d, J = 8.5 Hz, 2H), 6.86 (d, J = 8.8 Hz, 4H), 5.58 – 5.45 (m, 2H), 3.81 (s, 6H), 1.76 (t, J = 7.2 Hz, 12H).^{13}C NMR (126 MHz, Chloroform-d) δ 179.59, 156.21, 148.78, 140.53, 137.86, 133.12, 131.67, 131.09, 127.88, 126.86, 123.17, 120.34, 114.83, 113.12, 110.58, 55.52, 54.29, 54.03, 21.81, 21.77.

Synthesis of 1,6-di(5/6-(4-(bis(4-methoxyphenyl)amino)phenyl)-1,3-diisopropyl-benzimidazolium bromide)-hexane (4):

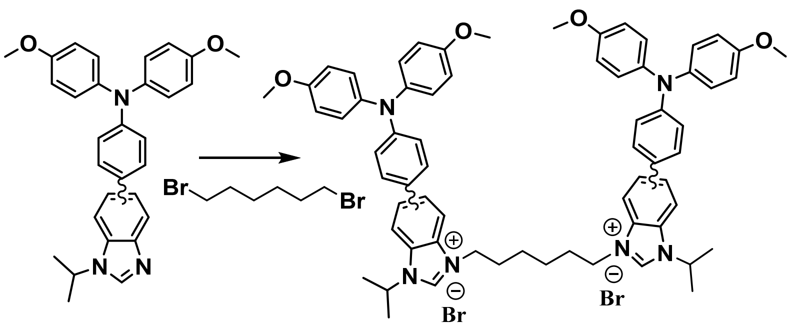

5/6-(4-(Bis(4-methoxyphenyl)amino)phenyl)-1-isopropyl-benzimidazole (0.20 g, 0.34 mmol, 1 eq.), 1,6-dibromohexane (0.04 g, 0.17 mmol, 0.5 eq), and 2 mL of tetrahydrofuran (THF) were mixed in a

pressure vessel. The reaction was heated at 120 °C and allowed to proceed for 48 hours. After completion of the reaction, the mixture was carefully transferred to a 100 mL round bottom flask and the solvent was removed by evaporation. The crude product was subjected to purification by column chromatography using a dichloromethane (DCM) and methanol (MeOH) mixture in a ratio of 10:1 as the eluent. The resulting white solid was collected with a yield of 56% (0.11 g). ^{1}H NMR (500 MHz, Chloroform-*d*) δ 11.29 (m, 2H), 7.92 – 7.78 (m, 2H), 7.73 (m, 2H), 7.64 (m, 2H), 7.37 (dd, *J* = 34.5, 8.0 Hz, 4H), 7.04 (d, *J* = 8.3 Hz, 8H), 6.94 (d, *J* = 7.8 Hz, 4H), 6.80 (d, *J* = 8.4 Hz, 8H), 4.94 (m, 2H), 4.71 (m, 4H), 3.74 (s, 12H), 2.16 (s, 4H), 1.86 – 1.73 (m, 12H), 1.63 (s, 4H).

Synthesis of 1,6-di(5/6-(4-(bis(4-methoxyphenyl)amino)phenyl)-1,3-diisopropyl-benzimidazole)gold bromide)-hexane (5):

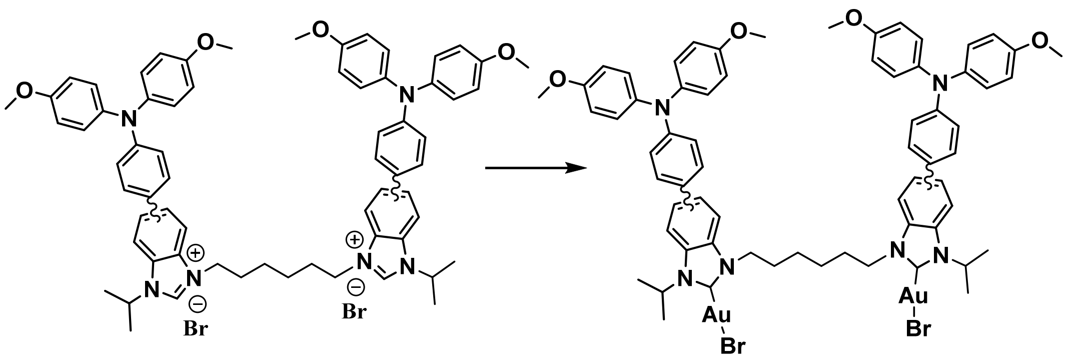

1,6-Di(5/6-(4-(bis(4-methoxyphenyl)amino)phenyl)-1,3-diisopropyl-benzimidazolium bromide)-hexane (0.10 g, 0.083 mmol, 1 eq.), $Au(SMe_2)Cl$ (0.05 g, 0.18 mmol, 2.1 eq.), K_2CO_3 (0.11 g, 0.83 mmol, 10 eq.), and 3 mL of anhydrous acetone were added to a two-neck round flask. The mixture was then heated to 65 °C and stirred overnight to promote the formation of the desired product. After cooling to room temperature, the solvent was evaporated, and the residual solid was dissolved in chloroform and filtered over Celite. The solvent was removed under vacuum and the crude product was purified by column chromatography using a gradient eluent (from pure DCM to DCM : MeOH= 100 : 1) to yield a white solid. (0.07 g, yield: 53%) ^{1}H NMR (500 MHz, Chloroform-d) δ 7.60 (m, 6H), 7.41 (dd, J = 23.5, 8.3 Hz, 4H), 7.10 (d, J = 8.4 Hz, 8H), 7.01 (d, J = 8.1 Hz, 4H), 6.86 (d, J = 8.3 Hz, 8H), 5.45 (dt, J = 14.0, 6.8 Hz, 2H), 4.52 (s, 4H), 3.81 (s, 12H), 1.99 (s, 4H), 1.75 (q, J = 6.9 Hz, 12H), 1.57 (s, 4H). ^{13}C NMR (126 MHz, Chloroform-*d*) δ 180.19, 156.16, 148.71, 140.58, 132.35, 127.96, 126.81, 123.91, 120.44, 114.82, 112.10, 110.33, 55.52, 53.78, 48.48, 29.69, 25.96, 21.91, 1.01.

Synthesis of gold nanoparticles with *N*-Heterocyclic Carbene Modified with Triphenylamines as Ligands (NHC-TPA@Au NP):

To a stirring solution of TPA-NHC-Au (I) complex (0.01 mmol) in $CHCl_3$ (2 mL), a freshly prepared solution of $NaBH_4$ (6 equiv., in 2 mL water) was added. The solution was stirred for 24 h, and then the red solution was evaporated to remove the solvents. The residues were washed with water (3*5 mL) and ethanol (3*5 mL). The Au NPs were further purified by centrifugation in a mixture of THF/EtOH (1/1) at 20 ° C.

The concentrated Au NPs were dried to obtain black solids, which were stored as solids at -20 ° C.

3. Experimental Data

Nuclear Magnetic Resonance (NMR) Spectroscopy

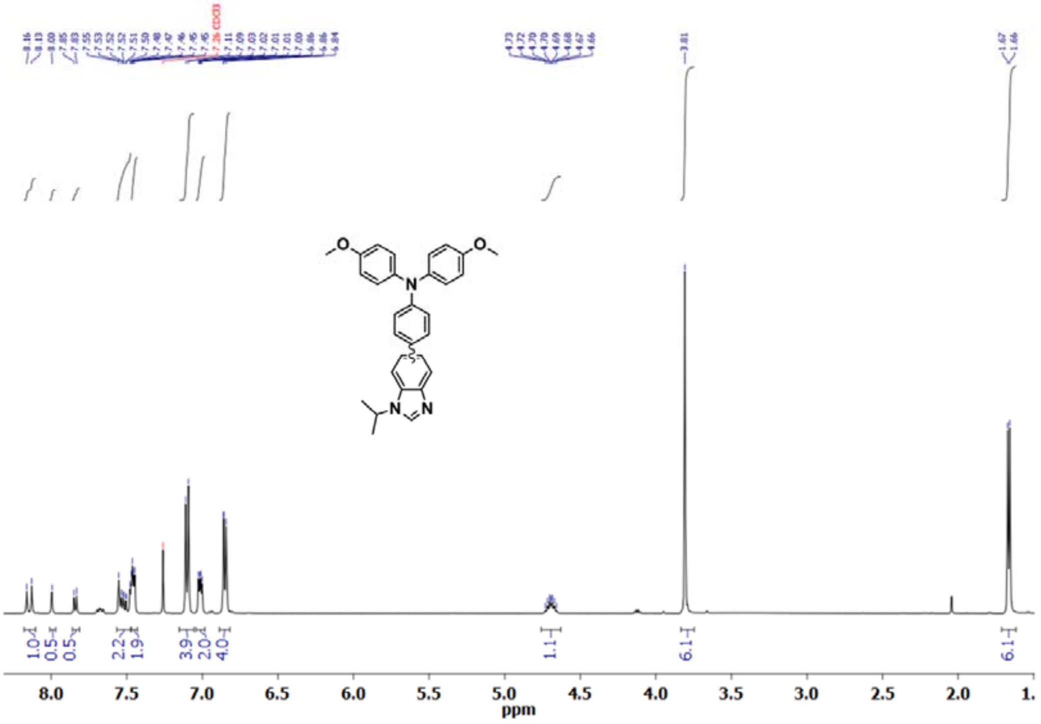

Figure S1: ^{1}H NMR spectrum of **1**.

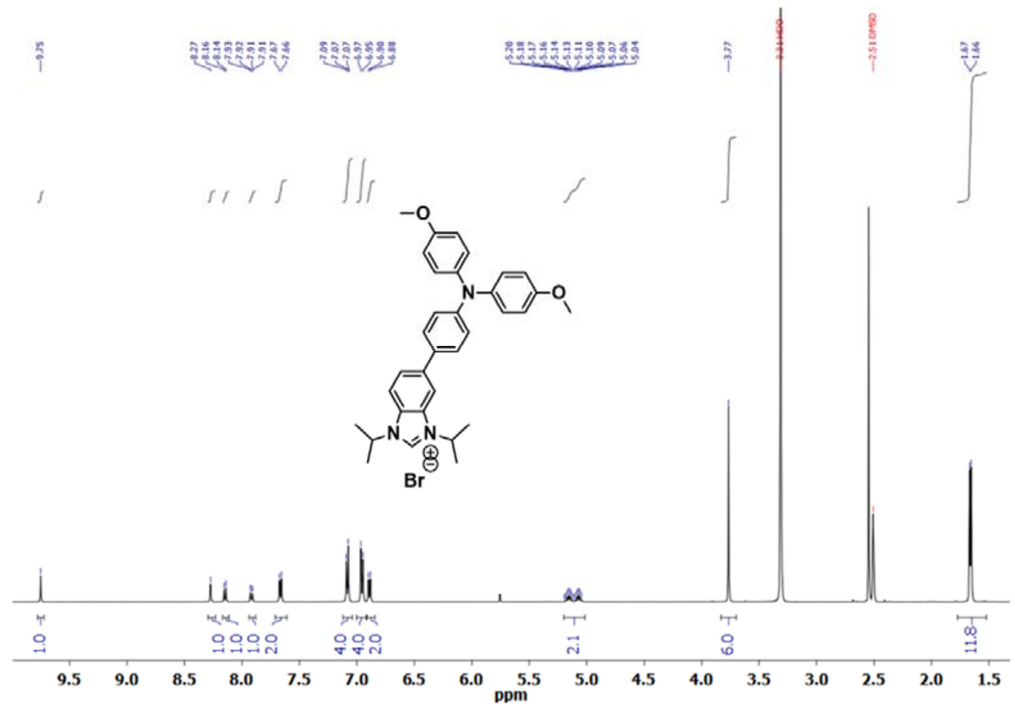

Figure S2: ^{1}H NMR spectrum of **2**.

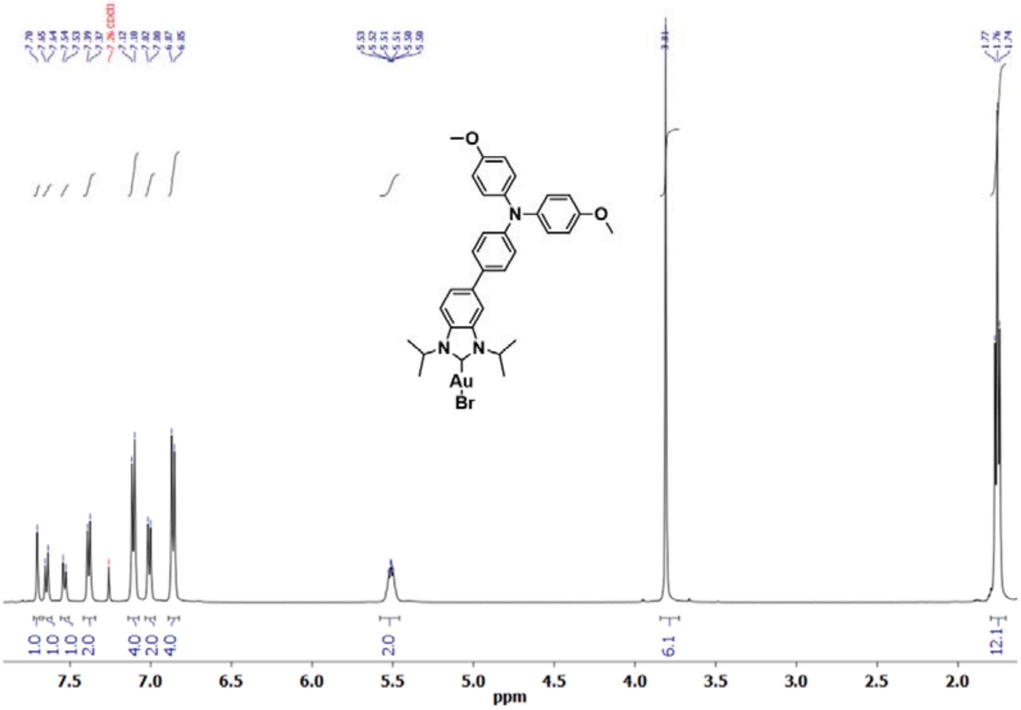

Figure S3: ^{1}H NMR spectrum of **3**.

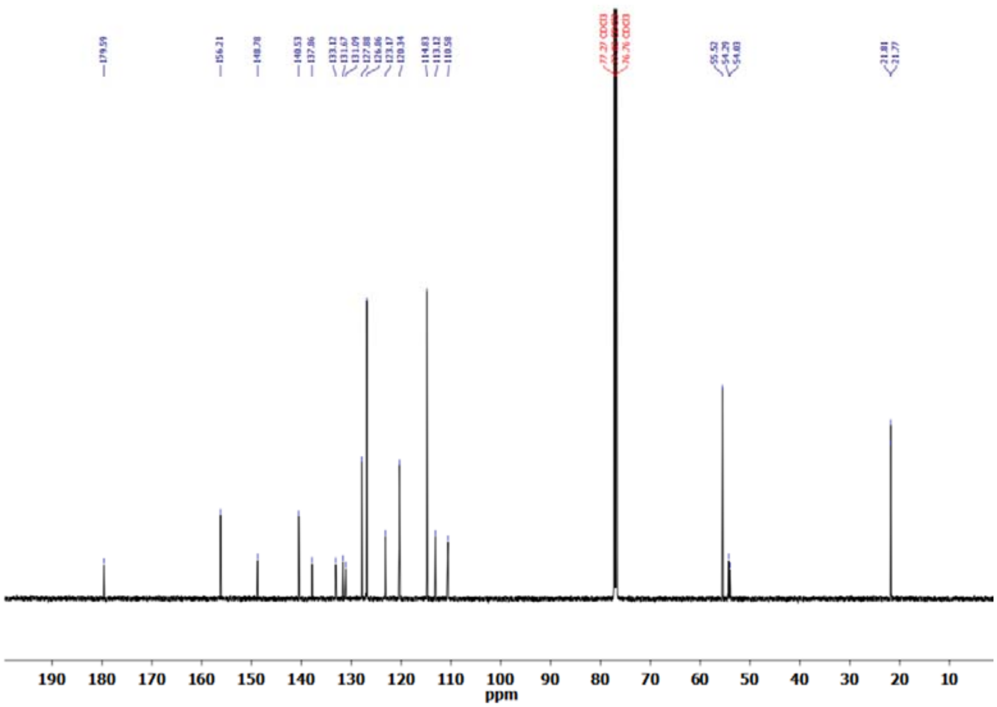

Figure S4: ^{13}C-NMR spectra of **3**.

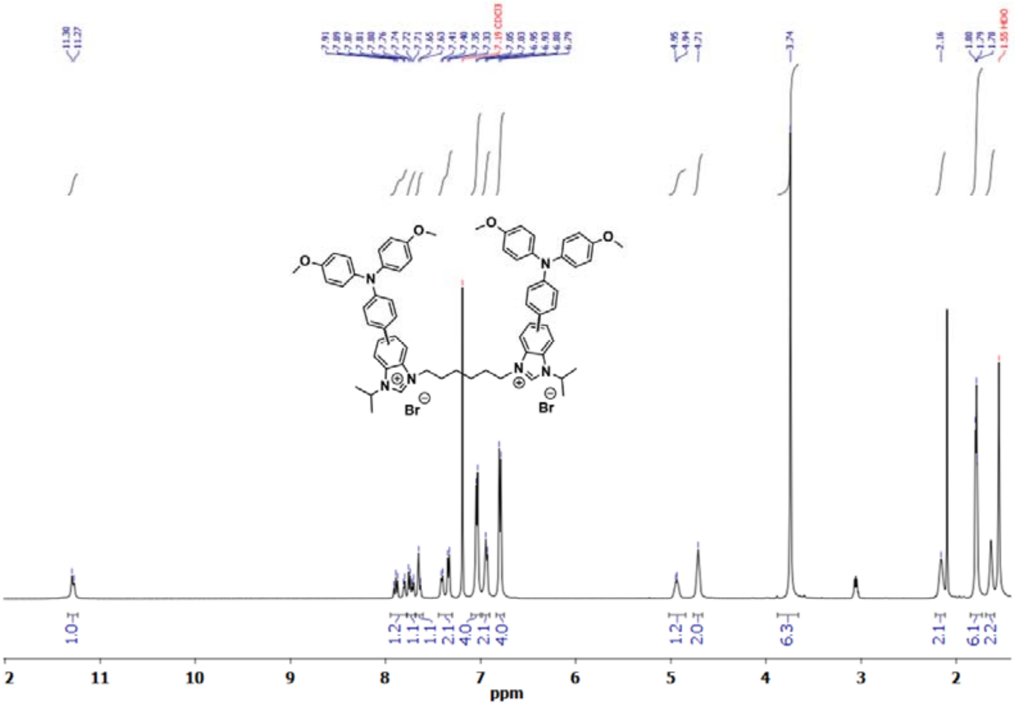

Figure S5: ^{1}H NMR spectrum of **4**.

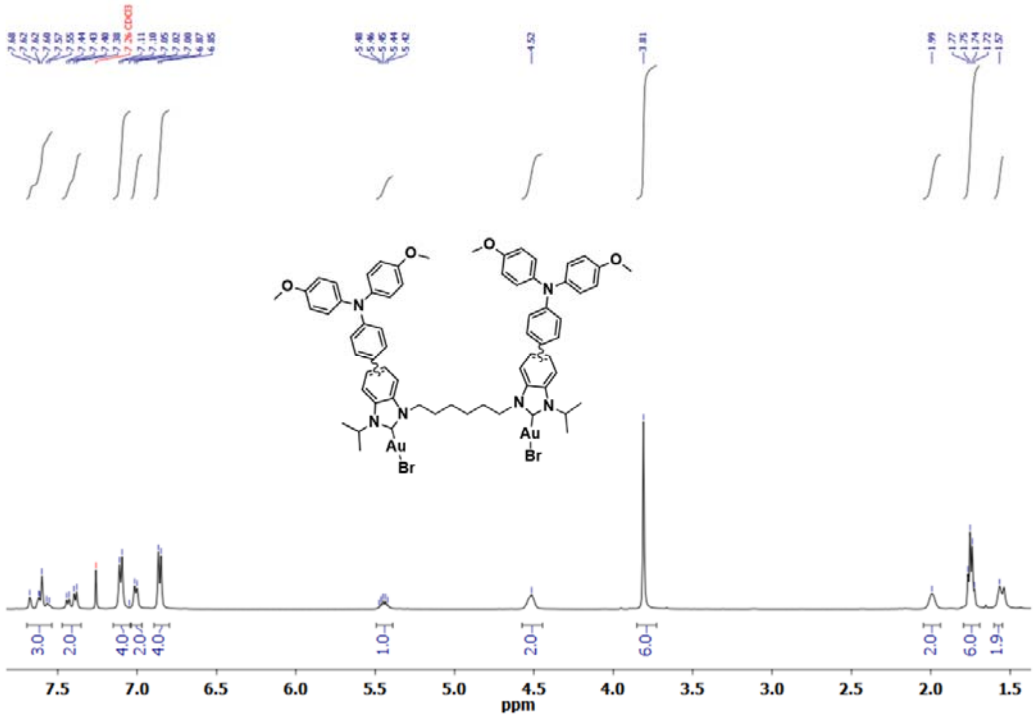

Figure S6: ^{1}H NMR spectrum of **5**.

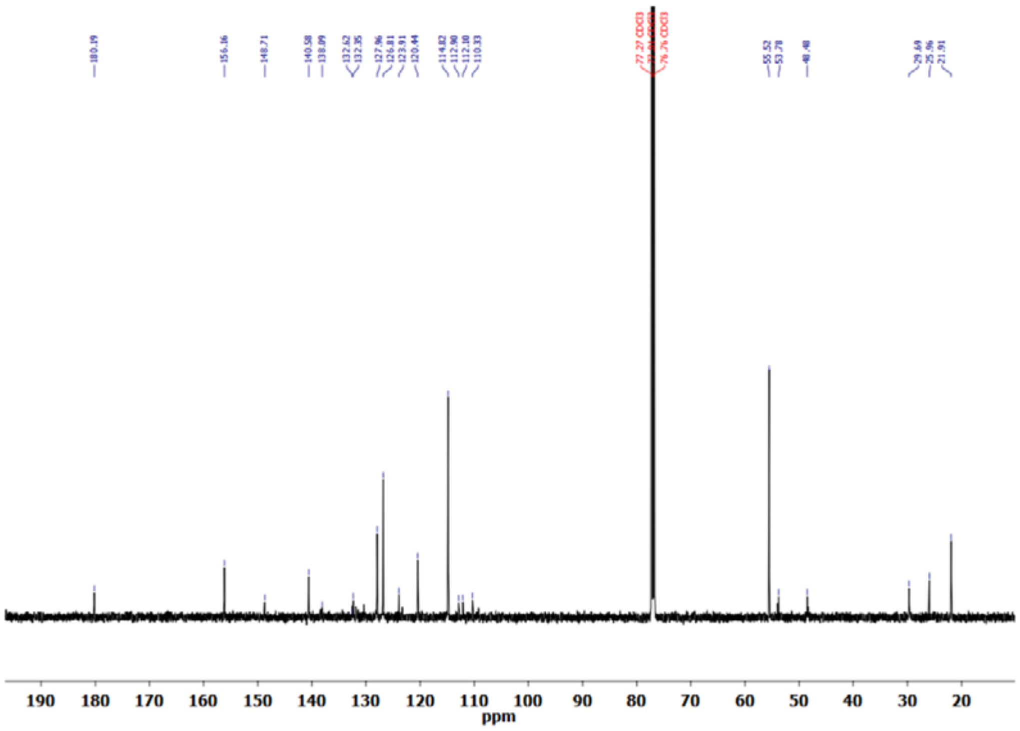

Figure S7: ^{13}C-NMR spectra of **5**.

XPS:

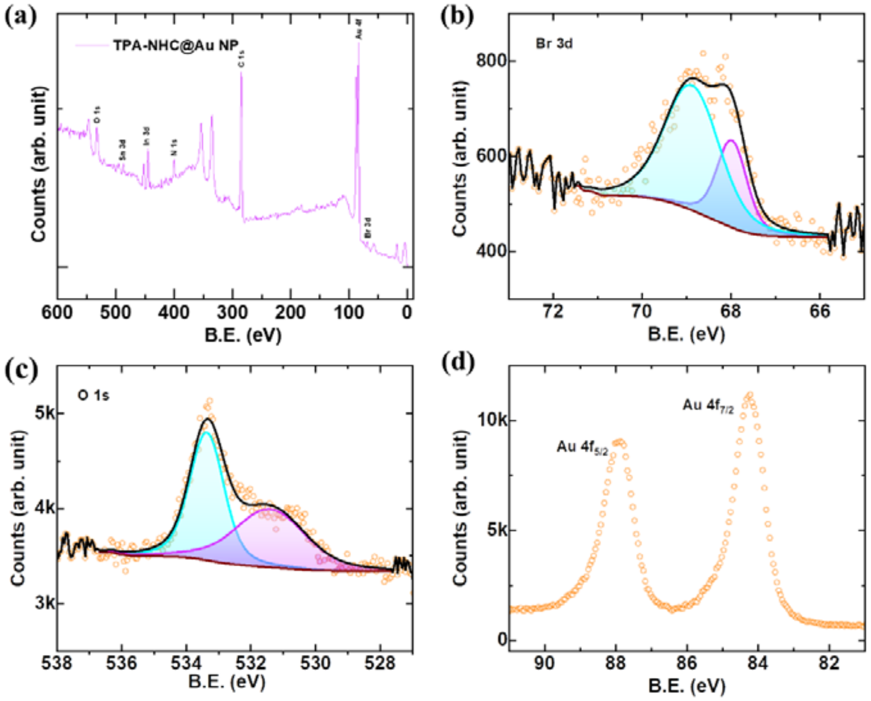

Figure S8: (a) XPS survey (b) Br 3d, (c) O 1s and (d) Au 4f core level spectra of TPA-NHC@Au NP.

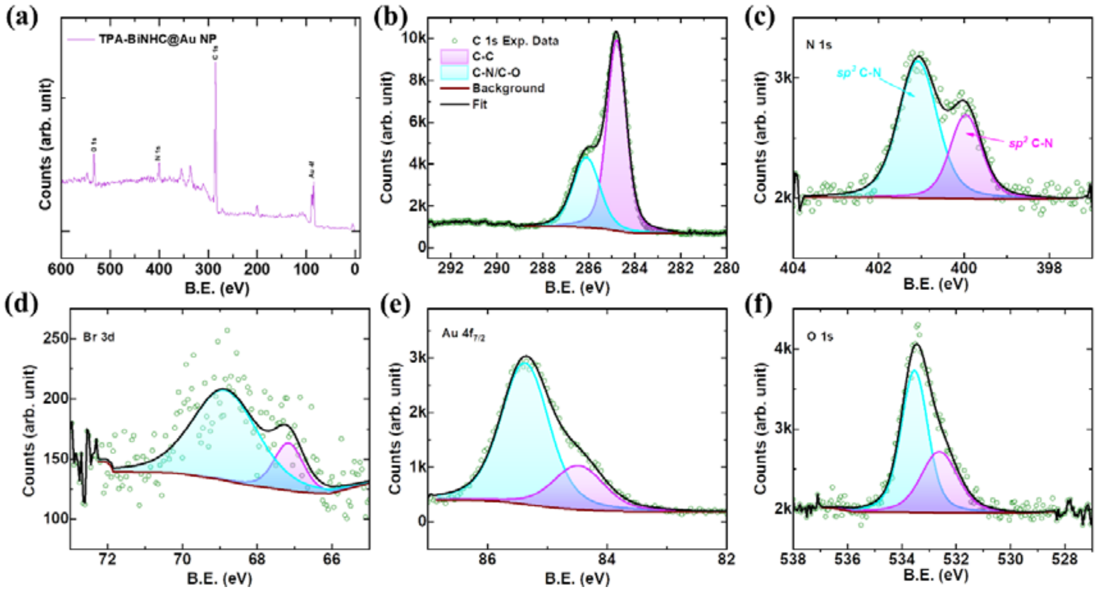

Figure S9: (a) XPS survey (b) C 1s, (c) N 1s, (d) Br 3d, (e) Au 4f and (f) O 1s core level spectra of TPA-BiNHC@Au NP.

Table S1: XPS atomic quantification for TPA-NHC@Au NP and TPA-BiNHC@Au NP.

Elements	TPA-NHC@Au NP (atomic %)	TPA-BiNHC@Au NP (atomic %)
Br 3d	1.46	0.41
Au 4f	6.37	1.65
C 1s	76.29	83.66
N 1s	6.39	6.86
O 1s	9.50	7.42

Thermal stabilities:

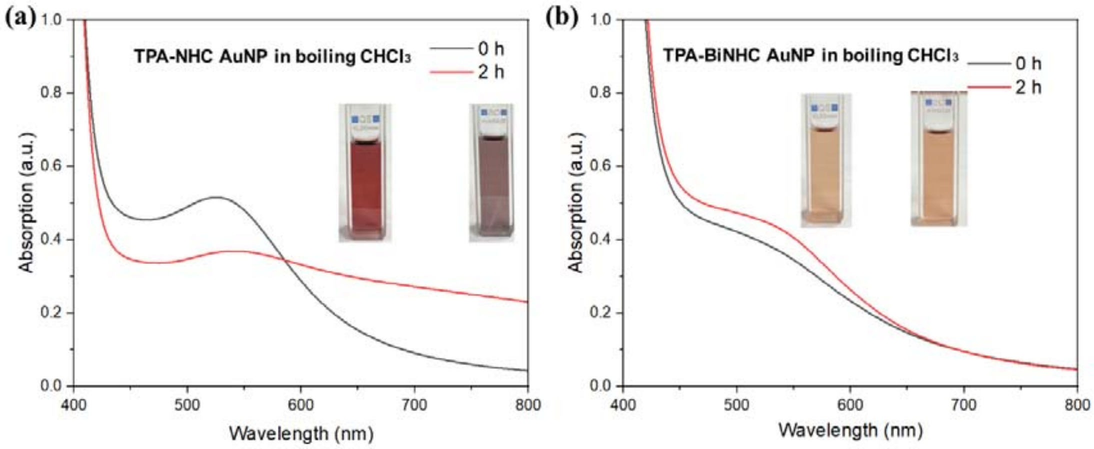

Figure S10: UV-vis absorptions of the (a) TPA-NHC@Au NP and (b) TPA-BiNHC@Au NP before and after in boiling $CHCl_3$ for 2 hours.

Chemical oxidation:

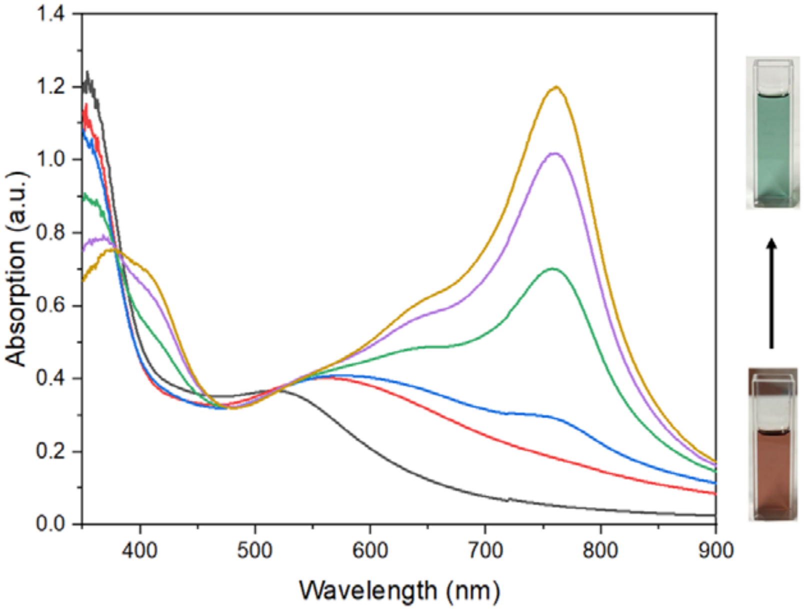

Figure S11: UV-vis absorption changes of the TPA-NHC@Au NP in $CHCl_3$ upon oxidation by adding Cu $(ClO_4)_2$ from the concentration of $1*10^{-4}$ to $6*10^{-4}$ M (inset shows the color changes from red to dark green).

Long-term electrochromic switching stability:

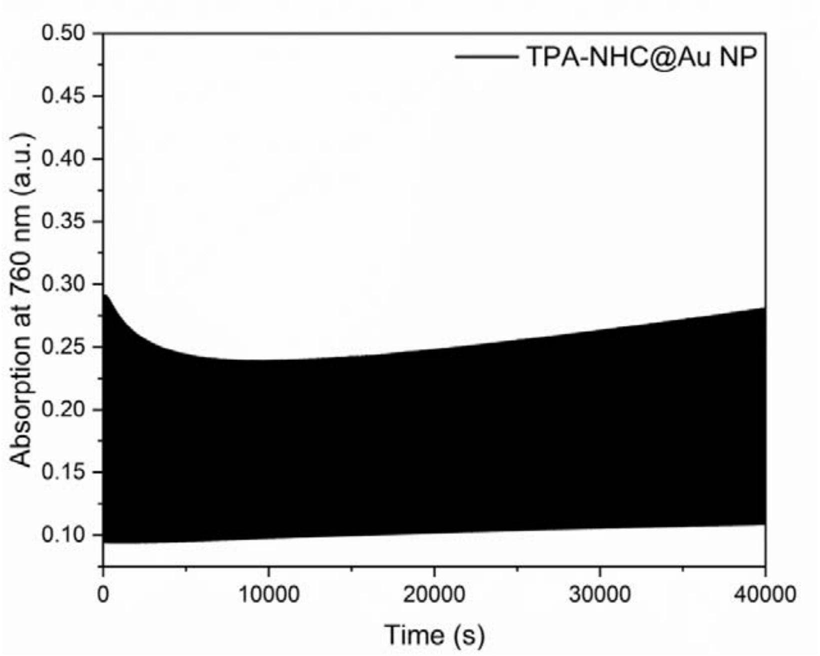

Figure S12: Switching stability of the TPA-NHC@AuNP-based electrochromic device for 1000

cycles.

AFM and C-AFM:

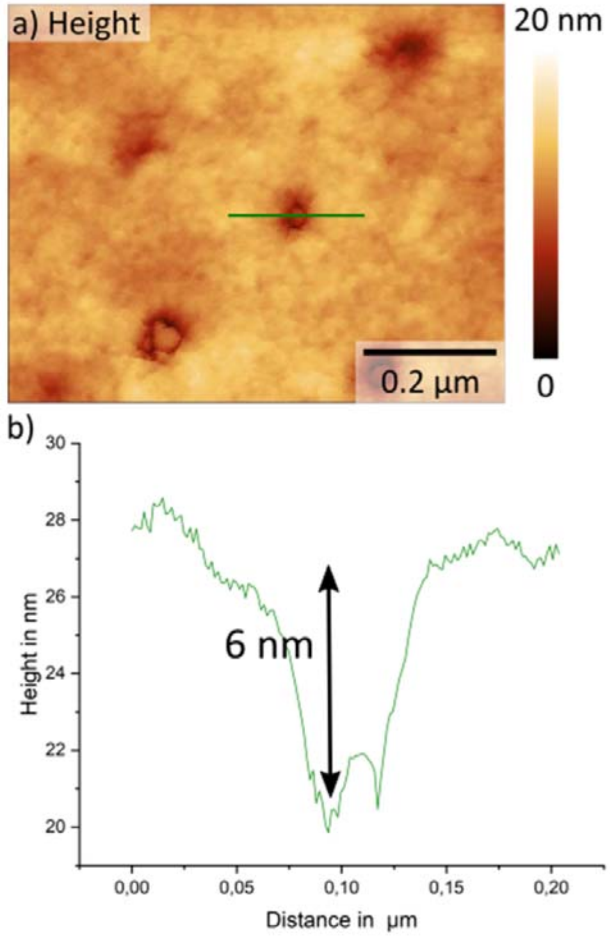

Figure S13: AFM image of TPA-NHC@Au NP (a) and the height profile taken across the grating lines (b).

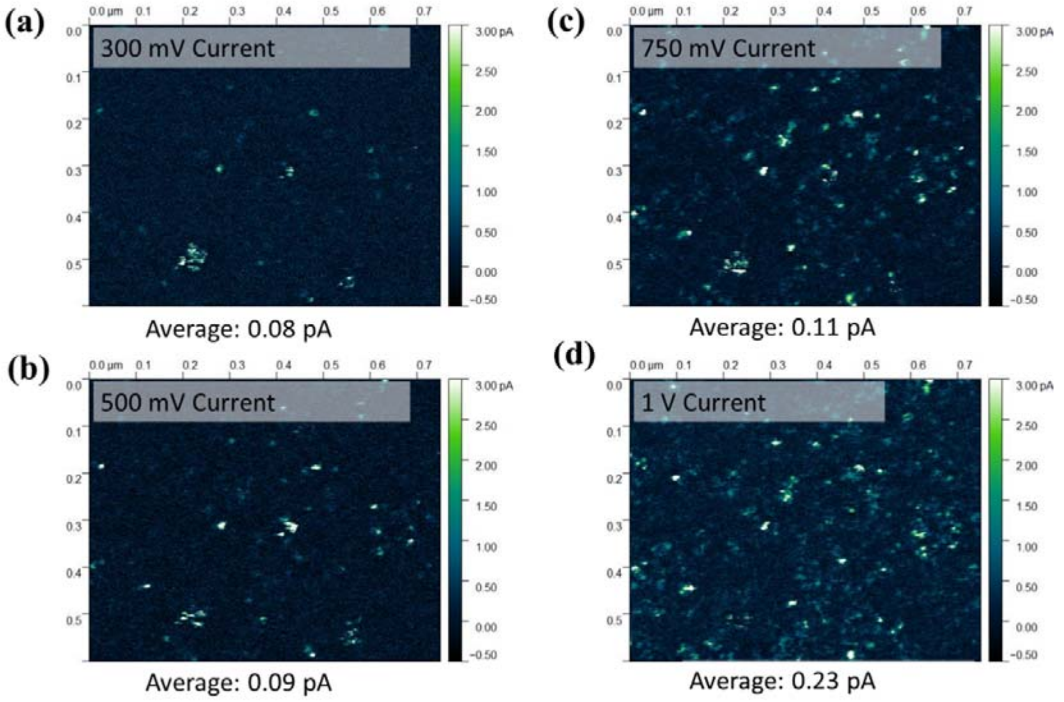

Figure S14: C-AFM images and contact currents under different bias at (a) 300 mV, (b) 500 mV, (c) 750 mV and (d) 1 V.

DFT calculation:

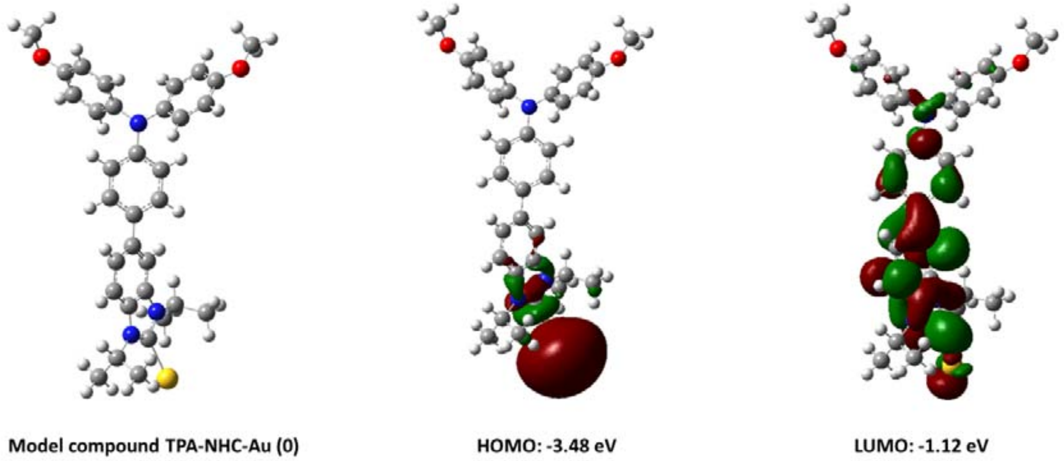

Figure S15: Molecular structure of TPA-NHC-Au (0), HOMO and LUMO distributions as well as energy levels derived from DFT calculations (B3LYP/6-31G(d)).

UPS and solar cells:

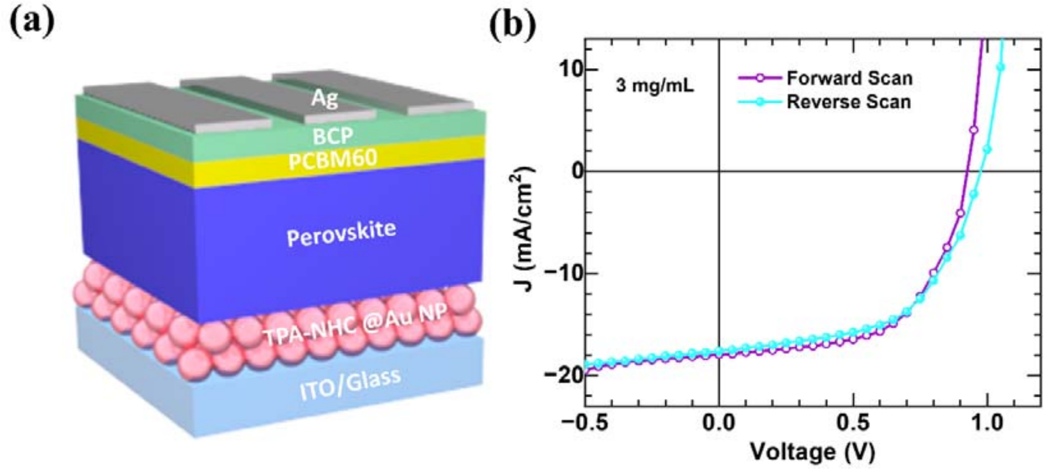

Figure S16: (a) The schematic diagram of the inverted architecture used to fabricate PSCs. (b) Illuminated J-V characteristic of PSCs with TPA-NHC@AuNPs as HTL.

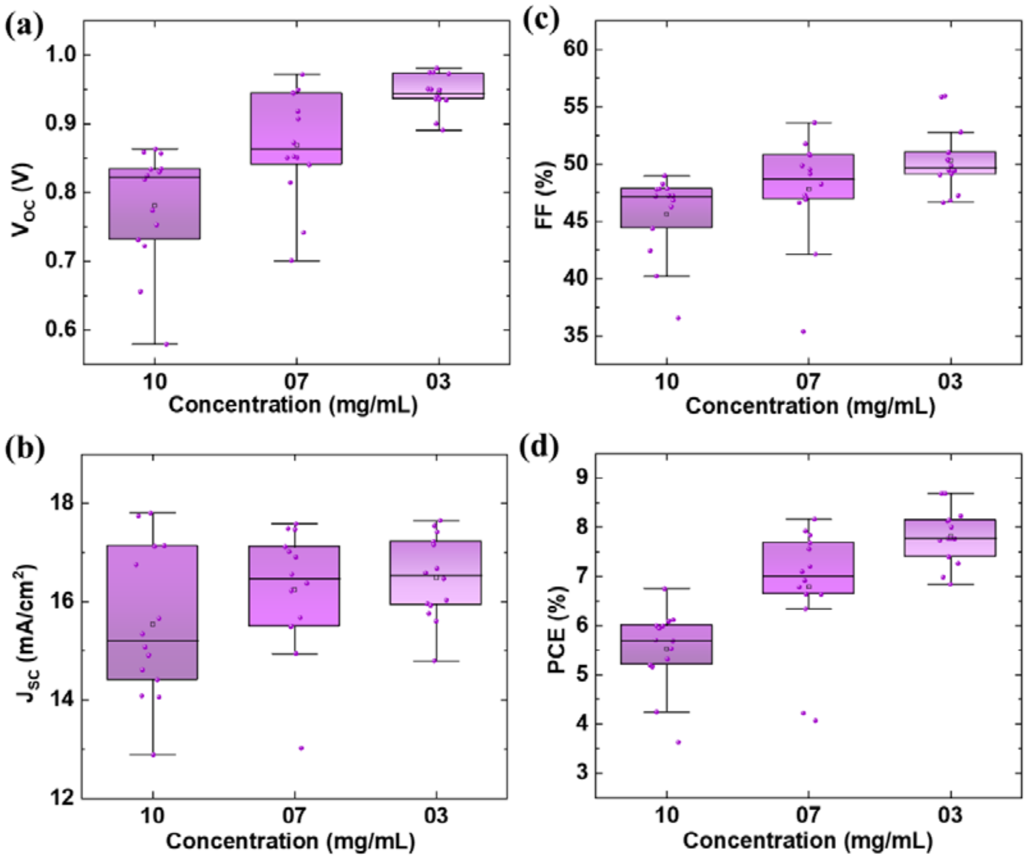

Figure S17: Photovoltaic parameters such as (a) VOC, (b) JSC, (c) FF and (d) PCE of an inverted architecture-based PSCs with NHC AuNPs as HTM. The PSCs were fabricated with three different concentration of NHC AuNPs in chloroform.
